# Case study: The influence of Achilles tendon rupture on knee joint stress during counter-movement jump – Combining musculoskeletal modeling and finite element analysis

**DOI:** 10.1016/j.heliyon.2023.e18410

**Published:** 2023-07-18

**Authors:** Zhenghui Lu, Dong Sun, Bálint Kovács, Zsolt Radák, Yaodong Gu

**Affiliations:** a Faculty of Sports Science, Ningbo University, Ningbo, 315211, China; b Research Institute of Sport Science, Hungarian University of Sport Science, Budapest, 1123, Hungary

**Keywords:** Achilles tendon rupture, Biomechanical simulation, Sports injuries, Finite element analysis, Knee joint contact force

## Abstract

**Background:**

Presently, the current research concerning Achilles tendon rupture repair (ATR) is predominantly centered on the ankle joint, with a paucity of evidence regarding its impact on the knee joint. ATR has the potential to significantly impede athletic performance and increase tibiofemoral contact forces in athletes. The purpose of this study was to prognosticate the distribution of stress within the knee joint during a countermovement jump through the use of a simulation method that amalgamated a musculoskeletal model of a patient who underwent Achilles tendon rupture repair with a finite element model of the knee joint.

**Methods:**

A male elite badminton player who had suffered an acute Achilles tendon rupture in his right leg one year prior was selected as our study subject. In order to analyze his biomechanical data, we employed both the OpenSim musculoskeletal model and finite element model to compute various parameters such as joint angles, joint moments, joint contact forces, and the distribution of knee joint stress.

**Results:**

During the jumping phase, a significantly lower knee extension angle (p < 0.001), ankle dorsiflexion angle (p = 0.002), peak vertical ground reaction force (p < 0.001), and peak tibiofemoral contact force (p = 0.009) were observed on the injured side than on the uninjured side. During the landing phase, the ankle range of motion (ROM) was significantly lower on the injured side than on the uninjured side (p = 0.009), and higher peak vertical ground reaction forces were observed (p = 0.012). Additionally, it is logical that an injured person will put higher load on the uninjured limb, but the finite element analysis indicated that the stresses on the injured side of medial meniscus and medial cartilage were significantly greater than the uninjured side.

**Conclusions:**

An Achilles tendon rupture can limit ankle range of motion and lead to greater joint stress on the affected area during countermovement jumps, especially during the landing phase. This increased joint stress may also transfer more stress to the soft tissues of the medial knee, thereby increasing the risk of knee injury. It is worth noting that this study only involves the average knee flexion angle and load after ATR in one athlete. Caution should be exercised when applying the conclusions, and in the future, more participants should be recruited to establish personalized knee finite element models to validate the results.

## 1 Introduction

Athletes commonly engage in sports activities that involve jumping, which subjects the Achilles tendon (AT) to significant stress. Overloading can lead to severe consequences, with the most detrimental being Achilles tendon rupture [1,2]. Even after undergoing rehabilitation, a significant proportion of athletes, approximately 30%, are unable to resume their previous level of performance [[3], [4], [5]]. Furthermore, the rupture can result in alterations in the morphology of muscles and tendons, consequently reducing free tendon stiffness [[6], [7], [8], [9], [10], [11], [12], [13], [14], [15], [16], [17]]. Additionally, muscle atrophy and changes in proprioception can also affect athletic performance [[18], [19], [20], [21], [22], [23], [24], [25]]. Even following Achilles tendon rupture repair (ATR), athletes still necessitate jumping and landing movements, thereby rendering it critical to understand the implications of repair for both athletes and coaches.

According to research findings, ATR has been shown to result in reduced posterior muscle strength and tendon length [10,12,13,17,19,26,27], which, in turn, may lead to a decrease in plantarflexion range and an increase in dorsiflexion range [[14], [15], [16],^28^]. Consequently, the individual's jumping ability may be negatively affected [24,25,29,30]. During high-intensity exercise, patients with ATR may compensate for ankle dysfunction by increasing knee flexion [[31], [32], [33], [34], [35], [36], [37]]. However, given the muscle weakness associated with ATR, such compensation may weaken or disrupt the knee joint kinetic chain, affecting pedal force and movement during landing [29,33,35,38,39]. It is also noteworthy that when performing jumping activities, using the injured limb may transfer joint stress from the ankle to the knee, thereby increasing the risk of knee injury [40]. Consequently, there is a crucial need to evaluate the impact of ATR on the knee joint functionality of patients.

Owing to the limited feasibility of directly measuring tibiofemoral forces, a commonly employed approach is to combine medical imaging, musculoskeletal modelling [41,42], and finite elements analysis [28,43]. However, relatively few studies have examined tibiofemoral forces during countermovement jump (CMJ) in patients with ATR [44]. As ATR can alter the length of the Achilles tendon, which, in turn, affect the length of the ankle moment arm and the estimated muscle forces, simulation-based approaches to estimate tibiofemoral forces during CMJ in ATR patients may offer theoretical guidance to researchers.

Previous research has found that patients with Achilles Tendon Rupture (ATR) exhibit bilateral lower limb asymmetry and greater knee joint moment and tibiofemoral force during movement and running [44,45]. However, the magnitude of knee joint medial-lateral contact force and internal stress during the countermovement jump (CMJ) process has not yet been fully determined. Therefore, this study aims to establish a lower limb musculoskeletal model based on medical imaging and kinematic data, to compare joint angles, moment, and tibiofemoral force of bilateral lower limbs during CMJ one year after surgery, and using a finite element model to calculate knee joint stress distribution. Based on previous research, we hypothesize that (a) due to ATR and degenerative changes in the triceps surae, and the ankle dorsiflexion angle will decrease, the plantarflexion angle will increase, but the plantarflexion moment will decrease; (b) compensatory knee joint phenomena will result in greater joint range of motion and peak extension moment in the injured side knee joint than in the non-injured side; (c) tibiofemoral force on the injured side will increase, leading to different medial-lateral and internal stress distribution in the knee joint compared to the non-injured side.

## 2 Methods

### 2.1 Participant

The male participant in this study was an elite badminton player who had experienced an acute rupture of his right Achilles tendon 12 months prior to the test. The participant was 25 years old, 1.73 m tall, and weighed 68 kg. He underwent open surgery for Achilles tendon repair within a week of the injury, a widely accepted treatment method for Achilles tendon rupture. During the surgical procedure, the attending physician made a small skin incision to expose the Achilles tendon directly and sutured the ruptured ends of the tendon together. The subject diligently adhered to the physician's instructions throughout the treatment process, wearing a plaster cast and ankle brace for the first month after the surgery. From the second month onwards, he was participating in physical therapy and rehabilitation exercises under the supervision of a physical therapist to restore the range of motion and strength of the ankle. The subject gradually increased the range and intensity of foot movements during the rehabilitation process, with the guidance of the physician and physical therapist. Prior to testing, the doctor confirmed the effectiveness of the surgery and rehabilitation through joint range of motion and pathological examination, and the subject had already resumed their daily specialized training. The study was performed in accordance with the Declaration of Helsinki and was approved by the Ethics Committee of Ningbo University (No. RAGH202205200004). A participant was informed of the experimental procedure and signed an informed consent form before testing.

### 2.2 Experimental design

To capture motion data, a total of 38 spherical reflective markers with a diameter of 12 mm were affixed to the participant's tights and skin using double-sided tape by an experienced researcher, following the guidelines of the Gait2392 musculoskeletal model. The marker trajectories were recorded using an 8-camera Vicon 3D motion capture system (Vicon Metrics Ltd., Oxford, United Kingdom), while ground reaction forces were simultaneously measured with an AMTI 3D force platform (AMTI, Watertown, Massachusetts, USA).

**Fig. 1:**
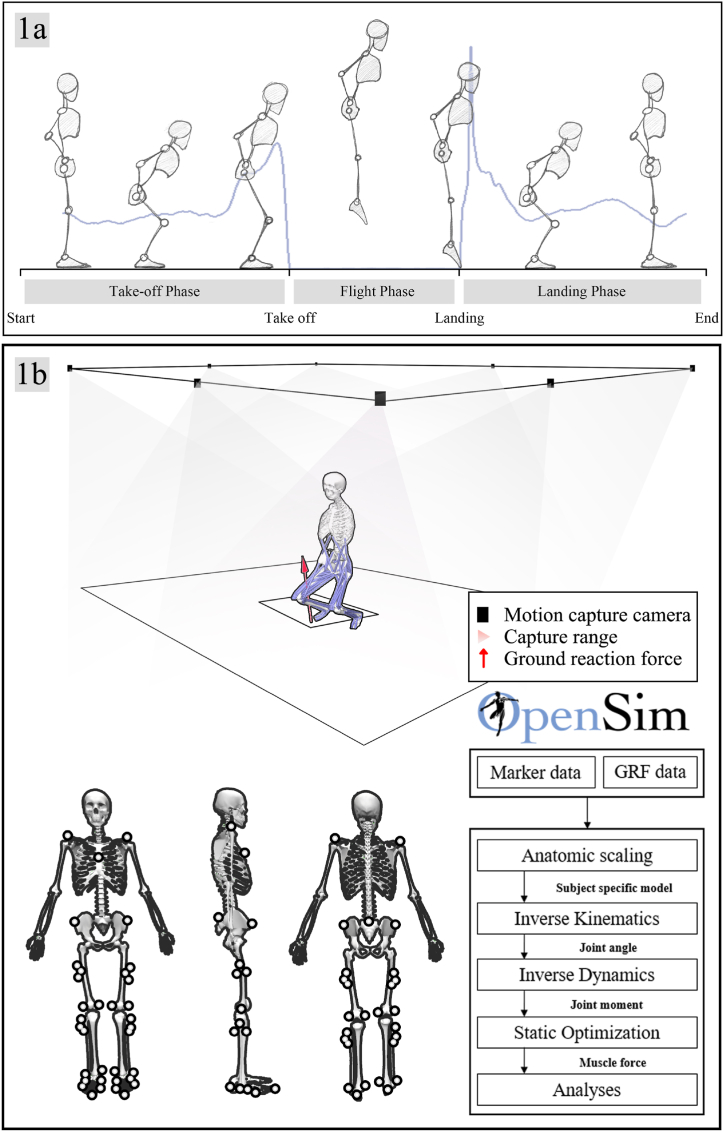
a, Schematic diagram of countermovement jump; b, Motion acquisition and OpenSim workflow.

Before the test, the participant completed a 5-min warm-up consisting of jogging and jumping at a self-perceived comfortable intensity. As shown in Fig. 1a, during the experiment, the participant stood on the force platform with arms akimbo, and the movement started from a fully extended knee position. Upon receiving instructions from the tester, the participant performed a brief countermovement to descend to a depth that felt most suitable to attain maximal jumping height, then executed a jump and endeavored to land in the same position as before the jump. The participant was allowed to cushion themselves during landing and return to the initial position. To prevent prolonging the flight by bending the legs [46,47], the participant had to jump as high as possible while maintaining hip, knee, and ankle extension throughout the flight. EMG signals from the rectus femoris, biceps femoris, anterior tibialis, and gastrocnemius muscles were acquired using a Delsys electromyography (EMG) test system (Delsys, Boston, Massachusetts, US) at 1000 Hz for model validation. To normalize the muscle EMG signal (0–100%), maximum voluntary muscle contractions were tested using the protocol recommended by Hall et al. (2019) [48]. Maximal gastrocnemius activity was assessed using a single leg heel lift position, maximal quadriceps activity was measured using a seated knee extension position, and maximal hamstring activity was measured using a prone knee flexion position. The raw EMG signals were initially filtered using a bandpass fourth-order Butterworth filter (10–500 Hz) in Delsys EMG Analysis software, and the root-mean-square (RMS) value of the EMG signal was calculated. EMG amplitudes were normalized to maximal voluntary isometric contraction (MVIC) activity for each muscle, and EMG activity was calculated on a scale from 0 (completely inactive) to 1 (fully active) by dividing the test RMS amplitude values by MVIC RMS amplitude values.

### 2.3 Musculoskeletal model

In this study, a modified Gait-2392 musculoskeletal model with 12 rigid body segments, 23 joint degrees of freedom, and 92 muscle-tendon actuators was utilized. The marker point trajectories and ground reaction force data obtained in the laboratory were converted to the formats required by OpenSim (.trc and.mot) using Matlab (MATLAB R2017a, MASS, Natick, MA, United States). As shown in Fig. 1b, the weights of the marker points in the model were manually adjusted to scale the model to match the participant's anthropometric characteristics based on anthropometric parameters and reflex markers affixed to body landmarks. To ensure accuracy, the RMS error between the marker points and virtual marker points in the experiment was less than 0.02 m, and the maximum error was less than 0.04 m. The muscle-tendon lengths and positions in the musculoskeletal model were manually adjusted from medical image to correspond to the participant's characteristics. Inverse kinematic were then applied to calculate joint angles and inverse dynamics was used to determine the joint net moment to minimize the error between the marked and virtual marked points in the experiment. The joint angles were defined as neutral joint angles when the participant's body was in anatomical position (i.e., the joint angle was 0°). Subsequently, a three-element Hill-type muscle model was used to estimate the degree of EMG activity and muscle forces during exercise using a static optimization algorithm that minimized muscle activation squared. Finally, the analysis tool was employed to compute tibiofemoral forces.

### 2.4 Finite element model

**Fig. 2:**
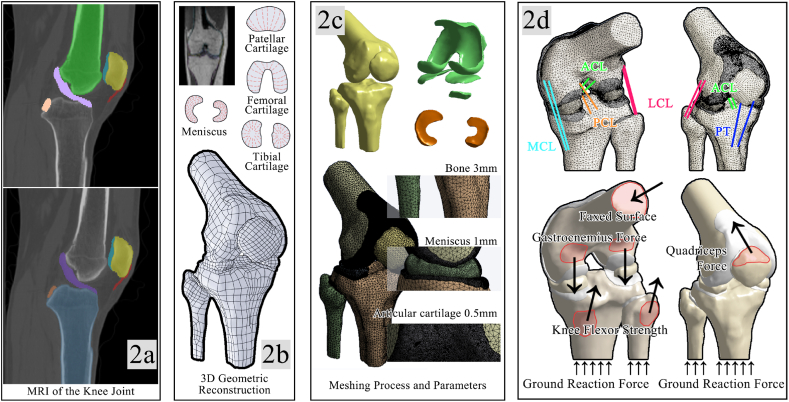
a, Mimics is used to perform image segmentation based on grayscale; b, Geomagic and SolidWorks are used to reconstruct the 3D model, adjust and smooth the model; c, Ansys Workbench is used to assign material properties to the model, partition the mesh; d, set up springs for calculation purposes.

The participant's knee was scanned by clinical magnetic resonance imaging (Philips 3T, General Electric, USA) while lying in a prone position with fully extended knee and neutral ankle position. The repetition time/echo time was set to 500/14 ms, the field of view was 20 cm and the slice thickness was 1 mm. The MRI images were imported into Mimics 21.0 (Materialise, Leuven, Belgium). As shown in Fig. 2, the borders of bones and soft tissues were determined using the image greyscale threshold segmentation method [49]. The resulting STL files were then imported into Geomagic Studio 2014 (Geomagic, Inc., Research Triangle Park, NC, United States) for smoothing and processed using SolidWorks 2021 (SolidWorks Corporation. MA, United States) to produce a solid model of the knee joint including the femur, tibias, fibulas, patella, and soft tissues. The finite element analysis software Ansys Workbench 2021 R1 (developed by ANSYS, Inc. located in Canonsburg, Pennsylvania, USA) was employed in this study for modeling and meshing. To ensure model accuracy and reliability, the tetrahedral meshing method was used to handle all solid parts during meshing. After conducting mesh convergence tests, the mesh sizes for the bone, enveloping soft tissue, and cartilage to 3 mm, 1 mm, and 0.5 mm, respectively. With these settings, the researchers developed a highly accurate and reliable model that serves as a solid foundation for subsequent simulation studies.

As shown in Fig. 2, the 3D finite element model of the knee joint was established to incorporate four bones, five cartilages, five ligaments, three muscle tendons, and the medial and lateral meniscus. To simplify the complexity of the model, all bone, cartilage, and meniscus materials are idealized as uniformly isotropic elastic materials.

The material properties of each tissue are shown in Table 1. As bone is considerably stiffer than other soft tissues, its effect was minimal in this study. Therefore, no distinction was made between cortical and cancellous bone. Articular cartilage primarily comprises a solid‒liquid biphase [50]. However, given the brief duration of the body's impact on landing, it can be assumed that no fluid flow occurs within the tissue under quick duration loading [51]. Therefore, the assumption of isotropic linear elasticity was used, which was sufficient to accurately predict the short-term cartilage response [52,53]. Similarly, the meniscus is assumed to be a monophasic linearly elastic isotropic material for the same reason. To simplify the calculation, the ligament was substituted with a bilinear spring. The relatively short toe region of the ligament suggests that the ligament is predominantly in the linear area during loading [54,55]. Thus, the bilinear elastic spring model was deemed acceptable [54,56].

**Table 1:** Model assumptions, material assignments and element types.

Structure		Number of elements	Model assumptions	Material assignment		
				Young's modulus (MPa)	Poisson's ratio	Tensile stiffness (N/mm)
Bones	Femur	82883	Isotropic linear elasticity	14200	0.30	–
	Tibia	68491				
	Fibula	10313				
	Patella	8718				
Cartilage	Femoral cartilage	125189	Isotropic linear elasticity	15	0.46	–
	Medial tibial cartilage	22168				
	Lateral tibial cartilage	21315				
	Patellar cartilage	51141				
	Tibiofibular cartilage	10604				
Meniscus	Medial meniscus	43251	Isotropic linear elasticity	27.50	0.33	–
	Lateral meniscus	27607				
Ligaments	Anterior cruciate ligament	–	Spring	–	–	380
	Posterior cruciate ligament					200
	Medial collateral ligament					100
	Lateral collateral ligament					100
	Patellar tendon					545

The proximal femur was fixed with the cartilage bound to the corresponding bone and the meniscus fixed to the tibial cartilage. Sliding friction was defined between the meniscus and the femoral cartilage, and between the cartilage of the pinna and the femoral cartilage, with a coefficient of friction defined as 0.04 [57,58], unless otherwise stated. The experimentally measured ground reaction forces were applied to the tibial and fibular cross-sections, while the forces of the gastrocnemius, biceps femoris, thin femoral muscle, semitendinosus and semimembranosus were applied to the internal and external superior femoral ankle, and the tibial and fibular planes of action. The surfaces of action of muscle forces on the bone were determined from the measured magnetic resonance images and further validated by using B mode ultrasound (Aloka, Tokyo, Japan) with a linear probe having a sampling frequency of 10 MHz and a distance resolution of 0.26 mm. Due to the transferability of the forces, the magnitude of the corresponding pressures applied to the cross sections of the tibia and fibula in the model was almost identical to the ground reaction forces. After defining the boundary and loading conditions, calculations were performed in Ansys Workbench 2021 R1 software with a generic hydrostatic algorithm. Von-Mises stresses and shear stresses on the meniscus and tibial cartilage were extracted.

### 2.5 Outcome measures

As shown in Fig. 1a, the CMJ motion was divided into two phases: the push-off phase and the landing phase, which spanned from the point of landing to the instant of attaining an upright stance. The ensuing biomechanical parameters were examined: 1) Knee and ankle joint angle and mobility. 2) Vertical ground reaction forces. 3) Knee and ankle joint moments. 4) Tibiofemoral joint contact forces. 5) Von-Mises stresses and shear stresses within the knee joint.

### 2.6 Statistical analysis

The present study analyzed pre- and post- CMJ movements of the participant. Raw data on hip-knee-ankle flexion/extension angles and moments, tibiofemoral joint contact forces, and medial knee contact forces were time-normalized (0–101 data points) for each participant. Due to the one-dimensional time series, one-dimensional statistical parametric mapping (SPM1D) paired sample *t*-test was used to compare joint kinematics and kinetics between the uninjured and injured sides. First, the scalar output was computed, and then the critical threshold (t*) was calculated, at which only α% of smooth random curves are expected to be traversed. If the *t*-test statistic trajectory crossed the critical threshold at any region or a point, the difference was deemed statistically significant.

Discrete value data were compared using paired samples t-tests. All statistical analyses were performed with MATLAB R2018a (MathWorks Inc., Natick, MA, USA) with a significance level of 5%.

## 3 Results

### 3.1 Achilles tendon length and model validation

**Fig. 3:**
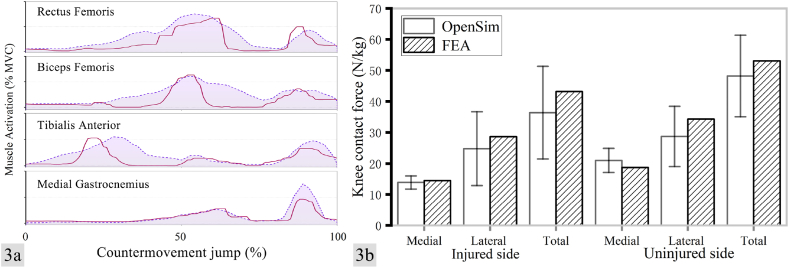
a, comparison of the degree of muscle activity obtained from OpenSim calculations with the EMG activity recorded from the experiment (including push-off, flight and landing phases); right; b, comparison of the medial and lateral tibiofemoral contact forces of the knee joint output from the finite element and musculoskeletal models (mean and SD).

Compared to the uninjured side, the injured side exhibited a 5.5% increase in slack length of the Achilles tendon. Fig. 3 demonstrates that the muscle activation during CMJ, as computed by the OpenSim static optimization tool, is comparable to the experimentally recorded EMG activity, and the medial and lateral tibial cartilage surface contact forces in the finite element model fall within one standard deviation of the medial and lateral tibiofemoral contact force values generated by OpenSim for the bilateral knee joints of the lower limbs, thus highlighting the greater reliability of the OpenSim model and the knee FEM model data.

### 3.2 Kinematics

**Fig. 4:**
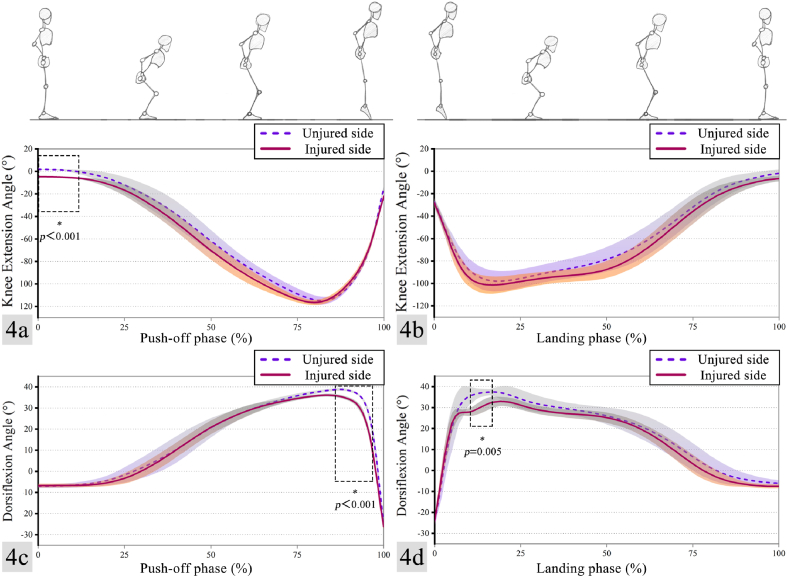
a, knee joint extension angle during vertical jump's push-off phase; b, knee joint extension angle during landing phase; c, ankle dorsiflexion angle during vertical jump's push-off phase; d, ankle dorsiflexion angle during landing phase. Blue dashed represents the mean of uninjured side, red solid represents the mean of injured side and the shaded area represents the standard deviation. (For interpretation of the references to color in this figure legend, the reader is referred to the Web version of this article.)

The knee and ankle joint angles of participant during the push-off and landing phases of the CMJ presented in Table 2 and Fig. 4. During the push-off phase of the CMJ, the injured side exhibited a significantly smaller peak knee extension angle compared to the uninjured side (t = 56.418, p < 0.001). The knee on the injured side remained flexed at the beginning of the movement (0%–12% of the push-off phase). No other statistically significant differences were observed in knee angle or range of motion (ROM) between the injured and uninjured sides at any other time during the CMJ. In addition, the magnitude of dorsiflexion angle on the injured side was significantly lower than that on the uninjured side during both push-off (t = 6.524, p = 0.002) (86%–97% of the push-off phase) and landing (t = 5.236, p = 0.002) (10%–17% of the landing phase). In addition, the injured side of the ankle exhibited less joint mobility during the landing phase (t = 3.834, p = 0.009).

**Table 2:** Flexion, extension angle and ROM of the knee and ankle joints.

Index	Injured side	Uninjured side	t-value	p-value
Max knee extension (°)				
Push-off phase	−4.75 ± 0.15	1.84 ± 0.21	56.418	0.000
Landing phase	−6.58 ± 2.48	−1.91 ± 3.51	3.489	0.013
Min knee flexion (°)				
Push-off phase	116.80 ± 2.37	115.12 ± 3.60	0.592	0.580
Landing phase	102.11 ± 7.55	98.57 ± 8.91	1.067	0.327
Knee joint ROM (°)				
Push-off phase	112.06 ± 2.45	116.96 ± 3.53	2.042	0.097
Landing phase	95.53 ± 7.07	96.66 ± 10.30	−0.151	0.885
Max dorsiflexion (°)				
Push-off phase	36.17 ± 0.71	38.83 ± 0.60	6.254	0.002
Landing phase	33.75 ± 1.81	39.35 ± 2.48	5.236	0.002
Max plantar flexion (°)				
Push-off phase	26.18 ± 1.11	24.89 ± 2.11	1.238	0.271
Landing phase	23.69 ± 1.81	23.89 ± 2.36	−0.249	0.779
Ankle joint ROM (°)				
Push-off phase	62.35 ± 0.79	63.72 ± 2.04	1.144	0.305
Landing phase	57.44 ± 1.60	63.24 ± 3.86	3.834	0.009

ROM, range of motion.

### 3.3 Kinetics

**Fig. 5:**
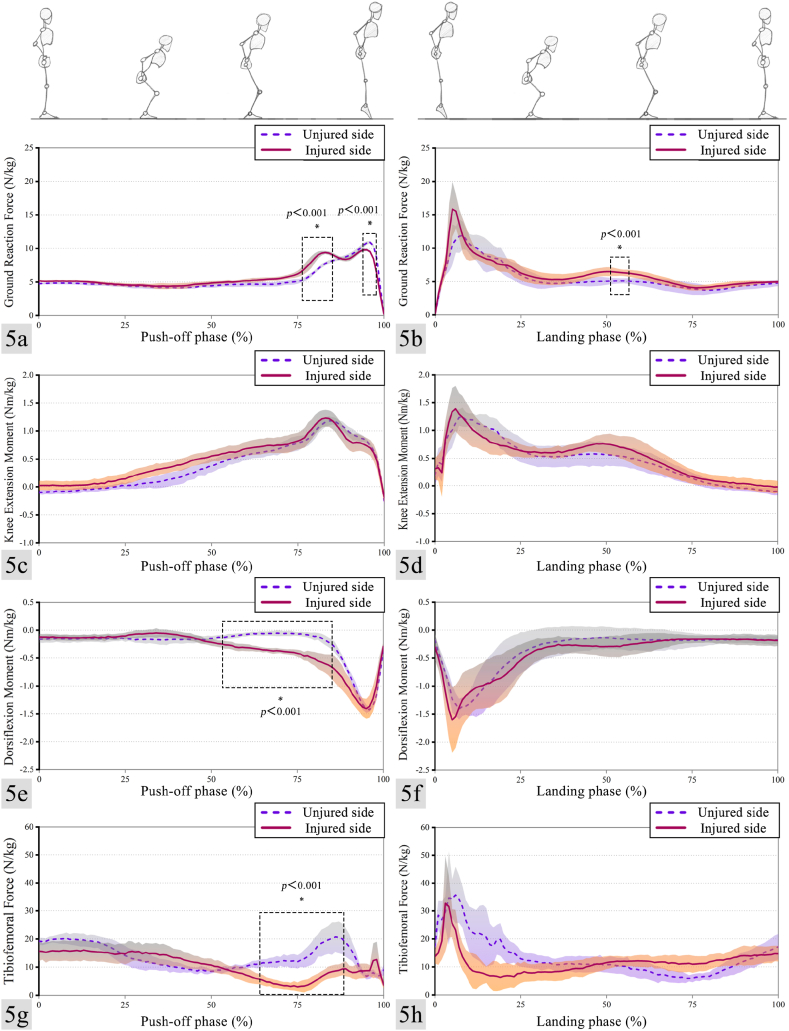
a, Vertical ground reaction force during push-off phase, b, Vertical ground reaction force during landing phase, c, Knee joint extension moment during push-off phase, d, Knee joint extension moment during landing phase, e, Ankle dorsiflexion moment during push-off phase, f, Ankle dorsiflexion moment during landing phase, g, Tibiofemoral contact force during push-off phase, h, Tibiofemoral contact force during landing phase. Blue dashed represents the mean of uninjured side, red solid represents the mean of injured side and the shaded area represents the standard deviation. (For interpretation of the references to color in this figure legend, the reader is referred to the Web version of this article.)

The vertical ground reaction forces, knee and ankle moments, and tibiofemoral joint contact forces of the participant during the push-off and landing phases of the CMJ are shown in Table 3 and Fig. 5. The injured side of the participant displayed significantly greater peak vertical ground reaction forces (t = 11.406, p < 0.001) and peak knee extension moments (t = −3.282, p = 0.022) during the push-off phase. In contrast, the peak tibiofemoral contact forces (t = 3.604, p = 0.009) were significantly lower than those observed on the uninjured side. Additionally, the disparities in kinetics between the injured and uninjured sides during the push-off phase were mainly evident at the termination of this phase. In addition, during the landing phase, there were on statistically significant differences in peak knee extension moment (t = −0.925, p = 0.386), peak plantar flexion moment (t = 1.021, p = 0.341), and peak tibiofemoral contact force (t = 1.499, p = 0.185) between the injured and uninjured sides. However, it is worth noting that the injured side demonstrated a significantly greater peak vertical ground reaction force during the landing phase (t = −3.391, p = 0.012).

**Table 3:** Vertical ground reaction forces, knee and ankle moments, and tibiofemoral contact forces.

Index	Injured side	Uninjured side	t	p
Peak vertical ground reaction force（N/kg）				
Push-off phase	9.84 ± 0.20	10.93 ± 0.20	11.406	0.000
Landing phase	18.90 ± 2.62	14.59 ± 2.13	−3.391	0.012
Max knee extension moment（Nm/kg）				
Push-off phase	1.25 ± 0.16	1.19 ± 0.07	0.992	0.335
Landing phase	1.54 ± 0.41	1.41 ± 0.10	−0.925	0.386
Max foot plantarflexion moment（Nm/kg）				
Push-off phase	1.41 ± 0.18	1.44 ± 0.05	−0.370	0.716
Landing phase	1.81 ± 0.43	1.59 ± 0.19	1.021	0.341
Tibiofemoral contact forces（N/kg）				
Push-off phase	19.44 ± 2.88	23.61 ± 1.19	3.604	0.009
Landing phase	48.21 ± 13.13	36.41 ± 14.94	−1.499	0.185

### 3.4 Finite element analysis

**Fig. 6:**
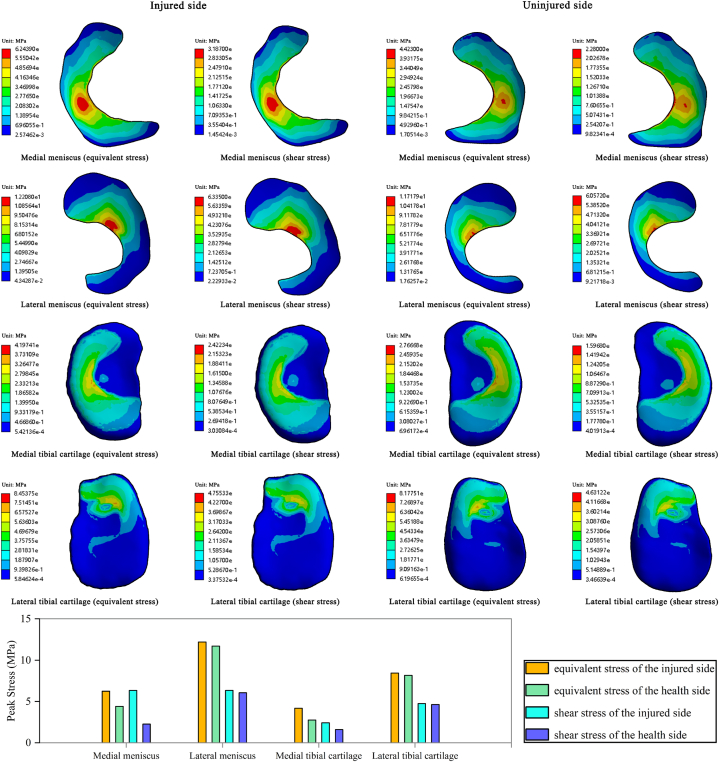
Von Mises stress, maximum shear stress distributions, and peak stresses in the meniscus and tibial cartilage. The change in color scaling represents the change in stress from large (red) to small (blue) on the stress cloud. (For interpretation of the references to color in this figure legend, the reader is referred to the Web version of this article.)

The examination of tibiofemoral contact forces revealed that the maximum forces on both sides of the participant transpired at around 4% of the landing phase. Moreover, an assessment of the stresses on the meniscal and tibiofemoral cartilage at peak tibiofemoral contact forces exhibited the distribution of Von Mises stresses, maximum shear stresses, and peak stresses on the injured and uninjured sides, which are displayed in Fig. 6.

The stress distribution areas on the medial and lateral menisci and tibial cartilage of the injured side did not exhibit a significant difference compared to those of the uninjured side during the CMJ landing, and the compressive stress distribution displayed a stress distribution pattern that was comparable to the maximum shear stress distribution. Analysis of the results revealed that peak von Mises stress (6.24 MPa on the injured side and 4.42 MPa on the uninjured side, 18.21%) and shear stress (3.19 MPa on the injured side and 2.28 MPa on the uninjured side, 28.46%) were amplified in the medial meniscus on the injured side compared to the uninjured side. Similarly, the peak von Mises stress (12.21 MPa on the injured side versus 11.72 MPa on the uninjured side, 4.01%) and shear stress (6.34 MPa on the injured side versus 6.06 MPa on the uninjured side, 4.39%) were elevated in the lateral meniscus on the injured side. Furthermore, the peak von Mises stress (4.20 MPa on the injured side versus 2.77 MPa on the uninjured side, 34.07%) and shear stresses (2.42 MPa on the injured side and 1.60 MPa on the uninjured side, 34.06%) were enhanced in the medial tibial cartilage on the injured side. The peak von Mises stresses (8.45 MPa on the injured side and 8.18 MPa on the uninjured side, 3.27%) and shear stresses (4.76 MPa on the injured side and 4.63 MPa on the uninjured side) in the lateral tibial cartilage on the injured side were also increased.

## 4 Discussion

Simulation provides a viable approach for biomechanical assessment, which obviates the requirement for invasive implantation of sensors. The latter approach is often challenging to apply and raises ethical concerns due to the risk of permanent damage. In this study, our aim was to evaluate the kinematics and joint moments of the knee and ankle joints, as well as the tibiofemoral joint contact forces during the countermovement jump (CMJ) in individuals with Achilles tendon rupture (ATR). To achieve this, we employed laboratory tests in conjunction with a customized OpenSim musculoskeletal model and finite element simulations.

AT ruptures can have long-term consequences for on athletes. Even after undergoing surgical intervention and rehabilitation, it remains critical to assess their potential effects on movement patterns, joint forces, and contact stresses in the lower limb joints (reference missing). AT rupture directly impair ankle function, leading to tendon lengthening and a reduction in plantarflexion strength [[59], [60], [61]]. Previous studies have predominantly examined the energy transfer between the ankle and other joints in the lower limb [11,28,44,62], which may contribute to an increased load on the knee joint, thus elevating the likelihood of knee injury during high-impact activities, such as running and jumping [[63], [64], [65], [66]]. Recent research by Powell et al. (2018) [62] and Willy et al. (2017) [11] both observe an increase in tibiofemoral forces among patients after AT rupture. However, little attention has been directed toward the knee joint in individuals with AT rupture, and the stress distribution within the knee joint remains uncertain.

Agres et al. (2015) [67] and Manegold et al. (2019) [16] reported a significant decrease in ankle sagittal range of motion (ROM) and an increase dorsiflexion angles on the injured side during walking among individuals with ATR. Similarly, Jandacka et al. (2017) observed a smaller ankle ROM and greater knee ROM during running in participants with AT rupture [68]. In contrast, our study showed that participants with AT rupture exhibited reduced ROM, dorsiflexion angle, and knee extension angle on the injured side during a single leg drop landing, which is inconsistent with our initial hypothesis. It is noteworthy that previous studies have primarily examined lower limb kinematics during walking or running. Willy et al. (2017) observed a smaller plantarflexion angle on the injured side during a single leg drop countermovement jump among individuals with ATR. Based on these findings, we hypothesize that, even after one year, AT morphology changes (such as longer slack length) may result in decreased CMJ performance and reduce the magnitude of ankle joint displacement. However, in contrast to walking, the injured side will actively reduce the degree of deformation of the elongated AT per unit time in a high-impact movements, such as jump, to mitigate the risk of re-injury [11]. Overall, current evidence on the changes in muscle-tendon interaction, load distribution, and joint mechanics resulting from ATR is limited, and further research is required to better comprehend these effects.

Some studies have investigated the biomechanical changes in the lower limb after AT injury, with most concentrating on walking or jogging [[68], [69], [70]]. The majority of these investigations have shown a decrease in ankle strength after AT rupture [11,24,71,72]. Moreover, elongation of the AT may cause a decline in ankle plantar flexion function during exercise [7], a decrease in dorsiflexion moment during walking [67], and an increase in knee joint work during running [68]. Notably, three studies examining the single-leg drop CMJ, Brorsson et al. (2017) [7], Powell et al. (2018) [62], and Willy et al. (2017) [11] observed that the knee contributed more significantly to total work than the ankle. These authors suggested that this movement pattern may compensate for reduced plantar flexor muscle function, but it may also elevate the risk of knee injury.

In this study, the participant executed a two-legged CMJ, and we categorized the movement phases as push-off and landing. Our outcomes demonstrated that during the jump phase, the affected side of the participant displayed significantly lower peak vertical ground reaction force, peak plantarflexion moment, and peak tibiofemoral contact force than the uninjured side. We also observed a minor increase in peak knee extension moment, which is consistent with previous research on single leg drop CMJ [7,11,62], and supports our research hypothesis. However, during landing, our results showed an opposite trend, with a significant increase in the peak vertical ground reaction force on the injured side. While there were no statistically significant differences in peak knee extension and plantarflexion moments, tibiofemoral forces were slightly greater on the injured side than on the uninjured side, potentially due to alterations in ankle range of motion (ROM). In some individuals with AT injuries, AT stiffness may persist higher than that of the uninjured side even years following surgery [67], contributing to the plantarflexion deficits and cause higher loads. Greater ankle ROM may increase the cushioning distance during exercise, mitigating excessive ground reaction force loading rates and peak ground reaction forces [73]. Based on our kinematic and kinetic results, we hypothesize that joint work may not transfer from the ankle to the knee during the landing phase, leading to increased joint loading due to ankle joint kinematic deficits.

Our musculoskeletal model was employed to predict the stress distribution within the knee joint during the landing phase. Our results indicated that the distribution of von Mises and shear stresses in the lateral tibial cartilage and meniscus of both the injured and uninjured knees did not differ significantly (within 5%) following the AT injury. However, the von Mises stresses and shear stresses in the medial tibial cartilage and meniscus were considerably higher on the injured side. The finding of the finite element analysis was consistent with the kinematic and kinetic data of the participants during landing, suggesting that as ankle mobility on the injured side decreased, peak ground reaction forces increased, resulting in a corresponding increase in internal knee stresses on the injured side, as we had hypothesized. It is reasonable to speculate that the mechanical changes caused by the elongation after ATR may lead to an imbalance in the internal and external knee stresses, particularly causing increase in the medial part on the injured side. This imbalance in stress distribution and stress concentration is closely related to soft tissue injuries, knee osteoarthritis, and other related diseases [74,75].

Many studies have been reported that AT defects or injuries may result in increased knee loading and an imbalance in medial and lateral knee contact forces [11,76,77]. However, to the best of our knowledge, this study is the first to investigate the distribution of internal knee joint stresses during CMJ maneuvers in athletes who have suffered from ATR. The personalized musculoskeletal model and finite element model developed for the knee in this study can aid in the evaluation of lower limb kinematics and kinetics of the lower limb after ATR and examination of stress distribution over the internal meniscus and tibial cartilage of the knee joint. Furthermore, these models can aid in exploring the relationship between degenerative changes in the muscles and knee pain experienced following ATR.

Although the model has been reasonably validated, caution must be taken in generalizing the conclusions to the entire population of Achilles tendon rupture patients, as the participants involved in this study represented only one athlete's average knee flexion angle and load (average muscle force and ground reaction force) after an ATR. In future studies, we recommend recruiting more participants and establishing personalized knee finite element models to validate our findings.

## 5 Conclusion

Currently, research on ATR still mainly focuses on the motion analysis of the ankle joint, lacking of evidence on the impact on the knee joint. Therefore, in this study, personalized musculoskeletal and knee joint finite element models were developed for a patient with unilateral Achilles tendon rupture based on experimental measurements and medical imaging data. The aim was to predict joint kinematics, joint moments, tibiofemoral forces, and knee joint internal stresses during CMJ. The findings indicate that during the landing phase, the injured side exhibited a lower ankle joint range of motion and greater vertical ground reaction force peak compared to the uninjured side. Moreover, tibiofemoral forces and medial knee joint stresses on the injured side were significantly greater than those on the uninjured side.

For athletes who undergo extensive training every day, even if the Achilles tendon is medically judged recovered, there is still a high risk of knee injury during high-intensity activities. It is worth noting that although the study had only one subject, the consistency of the results obtained can still be used by coaches or ATR patients to develop special training programs or rehabilitation plans. Based on the results of this study, we suggest that future research should increase sample sizes to confirm these findings, which would be of great significance for the ATR population.

## Author contribution statement

Zhenghui Lu: Conceived and designed the experiments; Performed the experiments; Analyzed and

interpreted the data; Wrote the paper.

Dong Sun, Kovács Bálint: Performed the experiments; Analyzed and interpreted the data.

Zsolt Radák: Analyzed and interpreted the data.

Kovács Bálint, Zsolt Radák, Yaodong Gu: Contributed reagents, materials, analysis tools or data; Wrote the paper.

## Data availability statement

Data will be made available on request.

## Declaration of competing interest

The authors declare that they have no known competing financial interests or personal relationships that could have appeared to influence the work reported in this paper.
